# *Aspergillus awamori* attenuates ochratoxin A-induced renal and cardiac injuries in rabbits by activating the Nrf2/HO-1 signaling pathway and downregulating IL1β, TNFα, and iNOS gene expressions

**DOI:** 10.1007/s11356-022-20599-y

**Published:** 2022-05-16

**Authors:** Doaa H. Assar, Samah Abou Asa, Moshira A. El-Abasy, Zizy I. Elbialy, Mustafa Shukry, Amera Abd El Latif, Mona N. BinMowyna, Norah A. Althobaiti, Mohammed A. El-Magd

**Affiliations:** 1 Clinical Pathology Department, Faculty of Veterinary Medicine, Kafrelsheikh University, Kafr El-Sheikh, 33516 Egypt; 2 Pathology Department, Faculty of Veterinary Medicine, Kafrelsheikh University, Kafr El-Sheikh, 33516 Egypt; 3 Poultry and Rabbit Diseases Department, Faculty of Veterinary Medicine, Kafr El-Sheikh University, Kafr El-Sheikh, 33516 Egypt; 4 Fish Processing and Biotechnology Department, Faculty of Aquatic and Fisheries Sciences, Kafrelsheikh University, 33516 Kafr El-Sheikh, Egypt; 5 Physiology Department, Faculty of Veterinary Medicine, Kafrelsheikh University, Kafr El-Sheikh, 33516 Egypt; 6 Department of Pharmacology, Faculty of Veterinary Medicine, Kafrelsheikh University, Kafr El-Sheikh, 33516 Egypt; 7 College of Applied Medical Sciences, Shaqra University, Shaqra, Saudi Arabia; 8 Biology Department, College of Science and Humanities-Al Quwaiiyah, Shaqra University, Al Quwaiiyah, 19257 Saudi Arabia; 9 Anatomy and Embryology Department, Faculty of Veterinary Medicine, Kafrelsheikh University, Kafr El-Sheikh, 33516 Egypt

**Keywords:** Ochratoxin A, *Aspergillus awamori*, Oxidative stress, Histopathology, Gene expression

## Abstract

Ochratoxin A (OTA) is one of the most dangerous and that pollute agricultural products, inducing a variety of toxic effects in humans and animals. The current study explored the protective effect of different concentrations of *Aspergillus awamori* (*A*. *awamori*) against OTA (0.3 mg/kg diet) induced renal and cardiac damage by exploring its mechanism of action in 60 New Zealand white male rabbits. Dietary supplementation of *A*. *awamori* at the selected doses of 50, 100, and 150 mg/kg diet, respectively, for 2 months significantly improved the rabbit’s growth performance; modulated the suppressed immune response and restored the altered hematological parameters; reduced the elevated levels of renal injury biomarkers such as urea, creatinine, and alkaline phosphatase; and increased serum total proteins concentrations. Moreover, it also declined enzymatic activities of cardiac injury biomarkers, including AST, LDH, and CK-MB. *A*. *awamori* alleviated OTA-induced degenerative and necrotic changes in the kidney and heart of rabbits. Interestingly, *A*. *awamori* upregulated Nrf2/OH-1 signaling pathway. Therefore enhanced TAC, CAT, and SOD enzyme activities and reduced OTA-induced oxidative and nitrosative stress by declining iNOS gene expression and consequently lowered MDA and NO levels. In addition to attenuating renal and cardiac inflammation via reducing IL-1β, TNF-α gene expressions in a dose-dependent response. In conclusion,this is the first report to pinpoint that dietary incorporation of *A*. *awamori* counteracted OTA-induced renal and cardiac damage by potentiating the rabbit’s antioxidant defense system through its potent antioxidant, free radical scavenging, and anti-inflammatory properties in a dose-dependent response. Based on our observations, *A*. *awamori* could be utilized as a natural protective agent against ochratoxicosis in rabbits.

**Graphical abstract:**

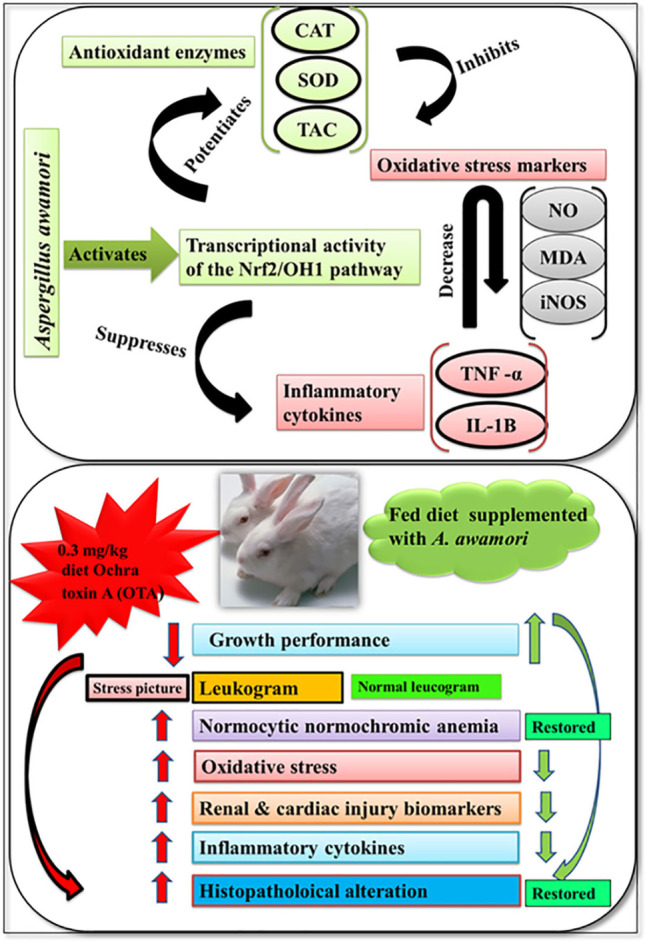

## Introduction

Ochratoxins are secondary toxic metabolites produced by various fungi of the genus Aspergillus and Penicillium (Ostry et al. 2013) that cause several harmful influences on multiple animals (Pfohl-Leszkowicz and Manderville 2007; Battacone et al. 2010). Rabbits are one of the applied alternative sources to face the scarcity of meat resources in developing countries (Dalle Zotte and Szendrő, 2011). Rabbit meat consumption is a rising economic industry routinely consumed in Egypt (Baviera-Puig et al., 2017). Rabbits are comparatively more vulnerable to Ochratoxin A (OTA) than mice, rats, and guinea pigs (Ponnuchamy, 2000). OTA has gained a special consideration among the recorded mycotoxins due to its nephrotoxic, teratogenic, embryotoxic, immunosuppressive, genotoxic, neurotoxic, and carcinogenic properties (O'Brien et al.^2001^). OTA has good thermal stability, making its eradication from the food chain impossible (Malir et al. 2016). Based on OTA nature, humans and domestic animals that are chronically exposed to low doses of OTA (50–250 μg OTA/kg b.m.) are potentially at risk for renal diseases or may even exhibit carcinogenic possibilities (Abdel-Wahhab et al. 2005; Ringot et al. 2006; Brown et al. 2007; Zain, 2011; Gruber-Dorninger et al. 2019). The most relevant impacts of OTA are the nephrotoxicity and nephron-carcinogenicity in rodents (Benford et al., 2001). The high sensitivity of kidneys to OTA can be attributed to the kidney^'^s critical importance as an exclusive excretory organ for OTA elimination (Marquardt and Frohlich, 1992).

Moreover, the exact mechanisms of its toxicity are not fully understood. Among these mechanisms, oxidative stress appears to be of particular interest as it is common for many toxic effects of OTA (Klauning and Kamendulis, 2004). Reactive oxygen species (ROS) have a significant role in mycotoxicosis, mediating cellular damage (Surai et al. 2008). ROS’s excessive formation leads to oxidative stress, which can trigger cell damage by oxidizing macromolecular structures and modifying their biological functions, ultimately causing cell cycle arrest and cell apoptosis (Ting et al. 2010). The nuclear factor erythroid 2-related factor 2 (Nrf2) is a transcription factor responsible for regulating cellular redox balance in eukaryotic organisms (Habtemariam, 2019). Once Nrf2 is activated, it binds to the antioxidant responsive elements (ARE) in the promoter region of target genes. Hence, activation of Nrf2 leads to the induction of HO-1 expression and other antioxidant proteins (Itoh et al. 2003). Antioxidant treatment is a medical approach for protection against the disturbed oxidant-antioxidant status and has been considered a hopeful remedy for the prevention and treatment of many diseases (Abd El Latif et al., 2021). For that, compounds that ameliorate OTA-induced disorders must be identified to be offered in animal diets and as protective agents for human health. Therefore, substantial efforts have been directed toward identifying natural antioxidants with free radical scavenging action to combat oxidative stress-mediated toxicity.

Feed supplements with immunostimulating properties as medicinal plants or probiotics have been widely used within the last years (Abdelhady and El-Abasy 2015; Abdelhady et al. 2017; Markowiak and Śliżewska 2018; Dawood et al. 2020 Moustafa et al. 2020; Assar et al. 2021). *Aspergillus awamori* has been known as a safe and efficient probiotic microorganism (Lee et al. 2008; Saleh et al. 2015). *A*. *awamori* supplementation in animal diet modulates digestive enzymes, therefore enhancing the nutrients digestibility (Tamang et al. 2016), reducing skeletal muscle lipid peroxidation (Saleh et al. 2012 and El-Deep et al. 2014), and improving the immune response of growing rabbits (El-Deep et al. 2021).

There are no previous reports on the modulating effects of *A*. *awamori* against induced renal and cardiac damage in rabbits. Therefore, the present study aimed to evaluate the protective effects of dietary supplementation with *A*. *awamori* against OTA-induced nephrotoxicity and cardiotoxicity in growing New Zealand rabbits by assessing the growth performance, hematological parameters, immune response, oxidative and antioxidant status, serum organ dysfunction biomarkers, histopathology with exploring the possible underlying regulatory molecular mechanism through gene expression profiles of *Nrf2*, *HO*-*1*, *iNOs*, *IL*-*1β*, and *TNF*-*α*.

## Materials and methods

### Chemicals

All chemicals used in this study were of analytical grade. Ochratoxin was purchased from Sigma Chemical Co. (St. Luis, MO, USA). Asp. Awamori powder was kindly supplied by Prof. K. Hayashi, Department of Biochemical Science and Technology, Faculty of Agriculture, Kagoshima University, Kagoshima, Japan. The kits of aspartate aminotransferase (AST), alkaline phosphatase (ALP), total protein, albumin, urea, creatinine, malondialdehyde (MDA), catalase (CAT), and total antioxidant capacity (TAC) were obtained from Biodiagnostics Co. (Cairo, Egypt). While LDH kit was purchased from Randox Laboratories Ltd. (Crumlin, UK), and CK-MB was obtained from Stanbio™ (CK-NAC [UV-Rate] kit; Boerne, TX, USA).

### Experimental animals and design

Sixty 6-week-old New Zealand male rabbits with an average body weight of 990 ± 5.05 g were obtained from Animal Production Research Center, Sakha, Kafr Elsheikh. Rabbits were acclimatized to a 16 h light/8 h dark cycle for 3 weeks before the experiment. They were individually housed in galvanized metal wire cages (60 × 50 × 35 cm) equipped with feeding and water troughs. Feed was formulated to cover all essential nutrient requirements for growing rabbits, as shown in Table 1 (De Blas and Mateos 1998; NRC 1997). Feed and water were offered ad libitum.

**Table 1:** Composition of the basic rabbit diet

Ingredients	%	Chemical analysis (% as DM)	%
Berseem hay	30.05	Dry matter (DM)	85.81
Barley grain	24.60	Crude protein (CP)	17.36
Wheat brain	21.50	Organic matter (OM)	91.42
Soybean meal (44% CP)	17.50	Crude fiber (CF)	12.37
Molasses	3.00	Ether extract (EE)	2.230
Limestone	0.95	Digestible energy (DE, kcal/kg)^(2)^	2412
Di-calcium phosphate	1.60	Calcium^(2)^	1.243
Sodium chloride	0.30	Total phosphorus^(2)^	0.808
Mineral-vitamin premix^(1)^	0.30	Methionine^(2)^	0.454
DL-Methionine Total	0.20 100	Lysine^(2)^	0.862

(1) One kilogram of mineral–vitamin premix provided: Vitamin A, 150,000 UI; Vitamin E, 100 mg; Vitamin K3, 21 mg; Vitamin B1, 10 mg; Vitamin B2, 40 mg; Vitamin B6, 15 mg; Pantothenic acid, 100 mg; Vitamin B12, 0.1 mg; Niacin, 200 mg; Folic acid, 10 mg; Biotin, 0.5 mg; Choline chloride, 5000 mg; Fe, 0.3 mg; Mn, 600 mg; Cu, 50 mg; Co, 2 mg; Se, 1 mg; and Zn, 450 mg

(2) Calculated according to** (**Mateos et al., 2010**)**

**Fig. 1:**
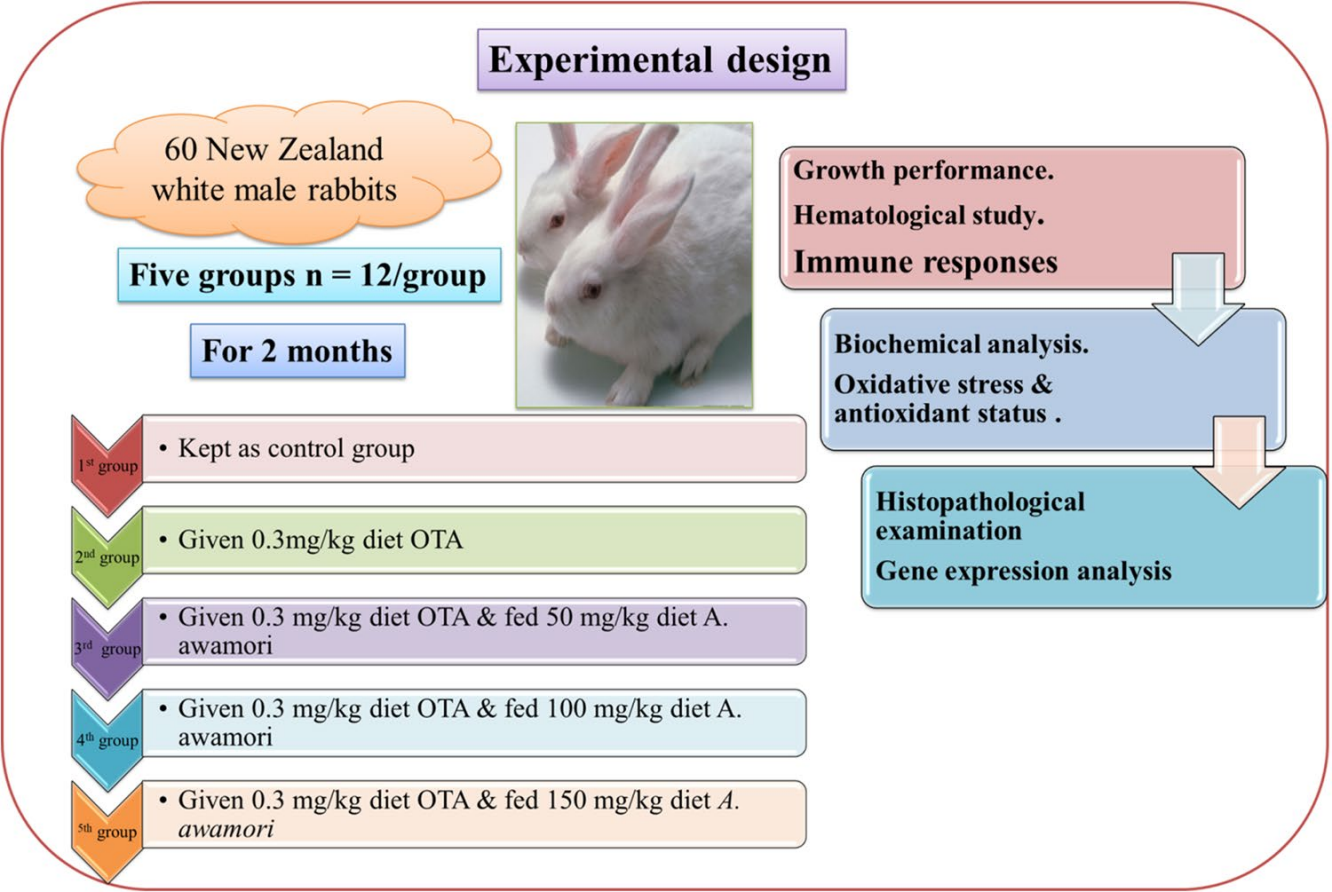
Experimental design

After the acclimatization period, rabbits were randomly divided into five groups (*n* = 12): the control group received a basal diet free from mycotoxins in the form of pellets; the OTA group received a basal diet contaminated with 0.3 mg/kg diet OTA following the dosage of (Prabu et al. 2013); OTA + *A*. *awamori* (group 3–5) fed basal diet contaminated with 0.3 mg/kg diet OTA and supplemented with 50, 100, and 150 mg/kg diet *A*. *awamori*, respectively following the dosage and formulation of Abdelhady et al. (2017). The treatments were administered for 2 months. The experimental protocol is described in (Fig. 1). Live body weight, feed intake, and several dead rabbits were recorded. The experimental protocol was approved and carried out following the Ethics Committee of Kafrelshiekh University.

### Formulation of OTA feed pellets

Fifteen milligrams OTA was dissolved in 100 ml ethanol and mixed thoroughly with a 50 kg diet to get 0.3 mg/kg OTA. The ethanol was then removed by vaporization under lowered pressure. Quantification of OTA in the diet was determined by reverse-phase liquid chromatography with post-column derivatization with iodine, as previously described by. The obtained analytical data were confirmed with solution fluorometry with bromine, and the deviations found ranged from 3.5 to 7.5%, with the best results obtained from the first quantification method. To validate this method in rabbit feed matrix, blank rabbit feed samples were provided for recovery determination. The mean recoveries for OTA was 85.5%, with mean relative standard deviations (RSD) of 16.3% as determined by reverse-phase liquid chromatography with post-column derivatization with iodine. The detection limits (LD) and quantification (LQ) were 0.3 µg/kg and 1.0 µg/kg, respectively.

### Sampling

At the end of the experimental period, rabbits were anesthetized (isoflurane, 5 mg/kg) (Parasuraman et al.^2010^). Blood samples were collected from the marginal ear vein of each animal. The 1st part was on EDITA-containing tubes for hematological assay, and the 2nd one was on the heparinized tube for estimating phagocytic activity. The 3rd part was collected on plain tubes left to clot at room temperature, then centrifuged at 3000 rpm for 15 min for serum separation and kept at – 20 °C for antioxidant assay and biochemical analysis. Then the rabbits were sacrificed, and kidney and heart samples were rapidly excised and collected from each animal for histopathological examination and real-time PCR.

### Growth performance and immunological assessment

Rabbits were weighed at the 6th and the 14th week of age, and the live body weights (LBW) in grams were recorded. Daily weight gain, feed intake, and feed conversion ratio (FCR) were calculated.

$$\begin{array}{c}\mathrm{Weight}\;\mathrm{gain}=\mathrm{Fina}\;\mathrm{lbody}\;\mathrm{weigth}\;\left(\mathrm g\right)-\mathrm{Initial}\;\mathrm{body}\;\mathrm{weigth}\;\left(\mathrm g\right)\\\mathrm{Feed}\;\mathrm{conversion}\;\mathrm{ratio}\;\left(\mathrm{FCR}\right)=\mathrm{Feed}\;\mathrm{in}\;\mathrm{take}\;\left(\mathrm g\right)/\mathrm{weight}\;\mathrm{gain}\;\left(\mathrm g\right)\end{array}$$

The performance index was calculated according to (Ayyat et al. 2018). The average body weight gain (BWG) and feed conversion ratio (FCR) were calculated according to Alagawany et al. (2016). Phagocytic activity (PA) and phagocytic index (PI) using were performed according to the methods of Rudkin et al. (2013).

Phagocytic activity = macrophages containing yeast/total number of macrophages × 100; Phagocytic index = number of cells phagocytized/Number of phagocytic cells.

### Hematological investigation

Red blood cells count (RBCs), hemoglobin (Hb) concentration and hematocrit value (PCV%), total leukocyte counts (WBCs), and differential leukocyte count were analyzed by using Orphee Mythic 22 CT Hematology Analyzer (Orphee SA Co., Plan-les-Ouates, Switzerland). Erythrocytic indices such as mean cell volume (MCV), mean cell hemoglobin (MCH), and mean cell hemoglobin concentration (MCHC) were determined as described by Feldman et al. (2000).

### Serum biochemical assay

Serum biomarkers for renal damage, including (urea, creatinine, sodium, potassium, and ALP), cardiac injury biomarkers such as (AST, LDH, and CK-MB) together with total proteins, albumin, and glucose, were determined. According to Larsen (1972) and Coulombe and Favreau (1963), creatinine and urea were determined. Total proteins (TP) were estimated according to Lowry et al. (1951), and albumin (Alb) was measured according to Henry et al. (1974). Alkaline phosphatase (ALP) was measured according to Tietz et al. (1983). According to Trinder (1951) and Terri and Sesin (1958), sodium and potassium were determined. Aspartate amino transferase (AST) was evaluated according to Reitman and Frankel (1957). Serum LDH activity was detected according to (Buhl and Jackson 1978). Creatine kinase MB (CK-MB) activity was measured according to the method developed by Szasz et al. (1979). Glucose was determined according to Trinder (1969).

### Estimation of lipid peroxidation and antioxidants biomarkers

Serum contents of lipid peroxidation biomarkers malondialdehyde (MDA) and nitric oxide (NO) were determined according to Esterbauer et al. (1982) and Montgomery and Dymock (1961), respectively. The enzymatic antioxidant biomarkers, including catalase (CAT), was estimated according to Aebi (1984); total antioxidant capacity (TAC) was determined according to Koracevic et al. (2001), and SOD according to Packer and Glazer (1990) following the manufacturers’ instructions (Bio-diagnostic Co., Giza, Egypt).

### Histopathological examination

The collected tissue samples from the kidney and heart of each animal were rapidly fixed in a 10% neutral-buffered formalin solution. Fixed specimens were processed through the standard paraffin embedding technique, including dehydration in ascending grades of ethanol, clearing in xylene, and embedded in paraffin wax. Then, 4-µm-thick paraffin tissue sections were stained with hematoxylin and eosin (H&E) (Bancroft and Layton 2013).

### Real-time PCR for gene expression of Nrf2, HO-1, IL-1β, TNF-α and iNOs

The real-time PCR (qPCR) was used to determine nuclear factor E2-related factor 2 (*Nrf2*), heme oxygenase-1 (*HO-1*), and inducible nitric oxide synthase (*iNOs*) in addition to inflammation-related genes; interleukin one beta (IL-1β), tumor necrosis factor-alpha (*TNF-α*). Total RNA was extracted from kidney and heart tissues of rabbits using Gene JET RNA Purification Kit (Thermo Scientific, # K0731, USA). RNA samples were reverse transcribed using Revert Aid H Minus Reverse Transcriptase (Thermo Scientific, USA, Cat no. EP0451) as previously described (Awad et al., 2020). The sequence of the used primers is presented in Table 2. In addition to primers and cDNA samples, 2X Maxima SYBR Green/ROX qPCR Master Mix (Thermo Scientific, USA, cat no. K0221) and StepOnePlus thermal cycler (Applied Biosystem, USA) was used to perform qPCR. The relative gene expression differences were normalized with the house-keeping gene *β actin*. The thermal cycling conditions included 10 min at 95 °C followed by 45 cycles (95 °C for 15 s, 60 °C for 30 s, and 72 for 30 s). Calculation of all fold changes in gene expression of treated groups (G2–G5) relative to expression in the control group (G1) was performed as previously depicted (El-Magd et al., 2016). Samples in each group were analyzed in triplicates and non-template control and negative RT controls in each plate.

**Table 2:** The used primers in qPCR

Gene	Forward (5^\^- 3^\^)	Reverse (5^\^- 3^\^)	Reference
IL-1β	CACCTCTCAAGCAGAGCACAG	GGGTTCCATGGTGAAGTCAAC	Badawy et al., (2018)
iNOs	GACCAGAAACTGTCTCACCTG	CGAACATCGAACGTCTCACA	Badawy et al., (2018)
TNFα	CTGCACTTCAGGGTGATCG	CTACGTGGGCTAGAGGCTTG	Schnupf and Sansonetti (2012)
HO-1	CCTCCCTGTACCACATCTACGT	AGCTCCTCCGGGAAGTAGAG	Levonen et al. (2007)
Nrf2	CACATCCAGACAGACACCAGT	*CTACAAATGGGAATGTCTCTGC*	Yamashita et al. (2014)
Β-actin	TCCTTCCTGGGCATGGAGTC	GGATGTCCACGTCGCACTTC	Attar et al. (2005)

### Statistical analysis

All data were examined for the normality and homogeneity by the Shapiro–Wilks test. The obtained data were analyzed using one-way ANOVA with Duncan’s post hoc test using SPSS version 17.0 (SPSS Inc, Chicago, USA) to determine the experimental groups. All data are presented as mean ± SEM. *P* value ≤ 0.05 was considered statistically significant.

## Results

### Clinical signs and post-mortem lesions

The control group was clinically normal, with no recorded mortalities. In contrast, OTA-treated rabbits showed loss of appetite, lethargy, and emaciation. Mortalities were recorded in 2 out of 12 rabbits (16.67%), which started from the 18th day until the 25th day. The central gross pathological lesion in dead and sacrificed rabbits who received OTA was the enlargement of their hearts and kidneys. Surprisingly, *A*. *awamori* supplemented rabbits were greatly improved clinically without mortalities in a dose-dependent response in all groups compared to OTA-treated rabbits. The best recovery was noticed in rabbits who received *A*. *awamori* at150 mg/kg diet*.*

### Growth performance and immune responses

**Fig. 2:**
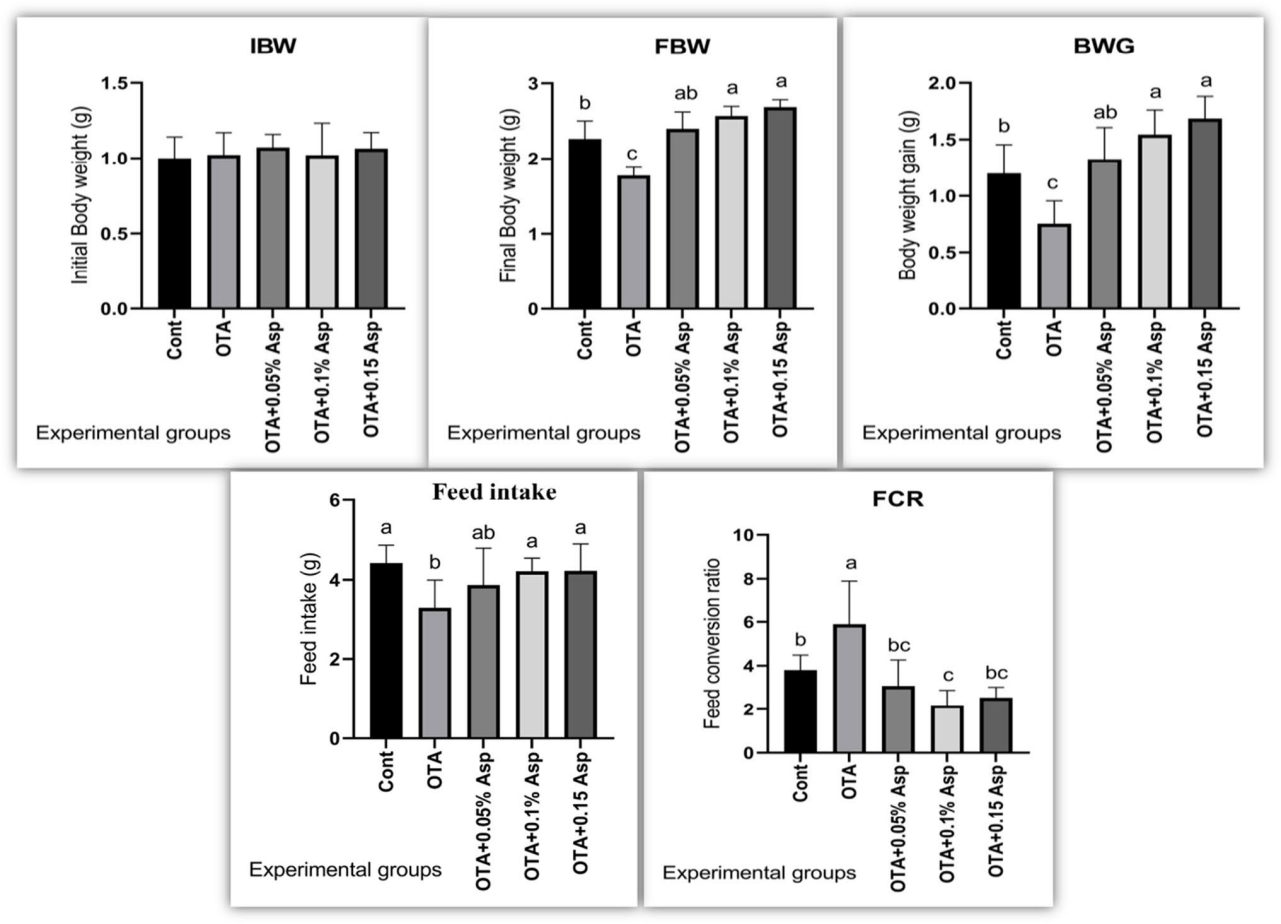
Effect of OTA and/or *A*. *awamori* supplementation on rabbits *IBW* initial body weight, *FBW* final body weight, *BWG* body weight gain, *FCR* feed conversion ratio. Data were presented as mean ± SEM (n = 7 per group). ^a–d^Means column carrying different superscript letters are significantly different at *P* ≤ 0.05

A significant reduction in feed intake, final body weight (FBW), and body weight gain (BWG), with a substantial increase in the food conversion ratio (FCR), was observed in rabbits fed 0.3 mg of OTA/kg diet compared to the control group (Fig. 2). However, rabbits fed an OTA-containing diet supplemented with *A*. *awamori* (50, 100, or 150 mg/kg diet) showed a significant improvement in feed intake, FBW, and BWG and a significant decline in FCR compared to the OTA treated rabbits. The improvement in growth performance was related to the increase in *A*. *awamori* concentration (Fig. 2).

**Fig. 3:**
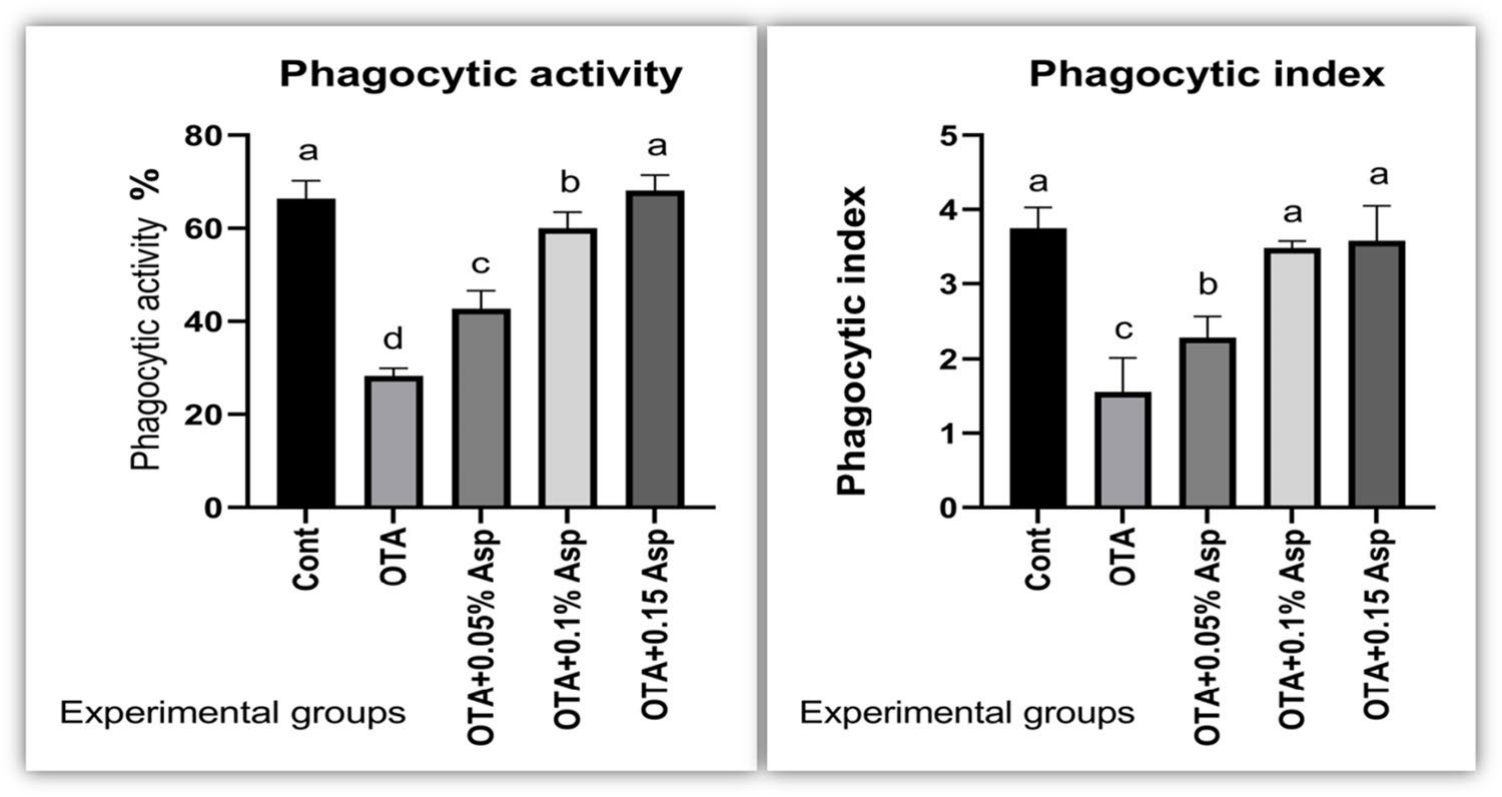
Effect of OTA and/or *A*. *awamori* supplementation on phagocytic activity and index. Data were presented as mean ± SEM (*n* = 7 per group). ^a–d^ Means column carrying different superscript letters are significantly different at *P* ≤ 0.05

Moreover, OTA-treated rabbits showed a significant reduction in the phagocytic activity (PA) and the phagocytic index (PI) compared to the control group. However, the dietary incorporation of *A*. *awamori* significantly enhanced the PA and the PI in a dose-dependent manner, with the best improvement by *A*. *awamori*’*s* highest concentration compared with the OTA treated group, as shown in Fig. 3.

### Hematological parameters

The OTA-intoxicated group displayed normocytic normochromic anemia as a decline in RBCs count, Hb concentration, and PCV% without changes in RBCs indices. In addition to stress, picture of leukogram is represented by a significant leukocytosis, heterophilia, monocytosis, lymphopenia, and eosinopenia. In addition to raised heterophils/lymphocytes ratio as compared to the control rabbits fed the basal diet (Table 3). In contrast, *dietary A*. *awamori* addition markedly restored the altered hematological parameters to the comparable references of the control rabbits. The best improvement was detected in rabbits who received *A*. *awamori* at a 150 mg/kg diet.

**Table 3:** Effect of OTA and/or *A*. *awamori* supplementation on hematological parameters of rabbits

Variable	Control	OTA	0.05% A. awamori	0.1% A. awamori	0.15% A. awamori
RBCs (× 10^6^/µl)	4.23 ± 0.24^a^	3.41 ± 0.16^b^	3.70 ± 0.13^b^	4.03 ± 0.09^a^	3.9 ± 0.09^a^
Hb (gm/dl)	8.25 ± 0.32^a^	7.32 ± 0.21^b^	7.64 ± 0.23^b^	7.93 ± 0.18^b^	8.12 ± 0.19^a^
PCV (%)	31.71 ± 0.92^a^	27.00 ± 0.83^b^	28.34 ± 0.76^b^	30.70 ± 1.21^a^	29.75 ± 0.36^a^
MCV (fl)	74.90 ± 5.80	79.18 ± 7.20	76.57 ± 0.93	76.18 ± 4.52	76.28 ± 2.52
MCH (pg)	19.50 ± 0.53	21.47 ± 1.82	20.65 ± 1.02	19.68 ± 0.65	20.82 ± 1.45
MCHC (%)	26.02 ± 1.70	27.11 ± 1.31	26.96 ± 1.14	25.83 ± 1.40	27.29 ± 1.40
WBCs (× 10^3^/µl)	7.87 ± 0.21^c^	12.88 ± 1.40^a^	11.78 ± 0.51^a^	10.55 ± 0.22^b^	9.11 ± 0.33^b^
Lymphocytes (10^3^/µl)	4.28 ± 0.16^b^	3.74 ± 0.33^c^	4.67 ± 0.15^a^	5.24 ± 0.14^a^	4.81 ± 0.16^a^
Heterophils (10^3^/µl)	2.83 ± 0.13^d^	7.99 ± 1.14^a^	6.05 ± 0.29^a^	4.39 ± 0.12^c^	3.57 ± 0.17^c^
H / L	0.66 ± 0.02^c^	2.14 ± 0.03^a^	1.29 ± 0.02^b^	0.84 ± 0.02^c^	0.74 ± 0.02^c^
Eosinophils (10^3^/µl)	0.21 ± 0.03^a^	0.04 ± 0.04^c^	0.08 ± 0.04^c^	0.18 ± 0.04^b^	0.19 ± 0.03^b^
Monocytes (10^3^/ µl)	0.55 ± 0.04^c^	1.11 ± 0.08^a^	0.98 ± 0.08^a^	0.74 ± 0.05^b^	0.54 ± 0.05^c^

Data were presented as mean ± SEM (*n* = 7 per group)

^a–d^Means within the same row carrying different superscript letters are significantly different at *P* < 0.05

*P* ≤ 0.05. All groups were compared to each other. *RBCs* red blood cells, *Hb* hemoglobin, *PCV* packed cell volume, *MCV* mean corpuscular volume, *MCH* mean corpuscular hemoglobin, *MCHC* mean corpuscular hemoglobin concentration, *WBCs* white blood cells

### Biochemical parameters

Rabbits fed OTA-contaminated diets showed significantly elevated levels of urea, creatinine, and ALP with a significant reduction in sodium, potassium, total proteins, and albumin levels compared to rabbits fed the basal diet only (Table 4). The levels of these biomarkers were markedly modulated and normalized in all rabbits fed a diet containing OTA and *A*. *awamori*, with the best-documented impact in rabbits who received *A*. *awamori* at 150 mg/kg diet.

**Table 4:** Effect of OTA and/or *A*. *awamori* supplementation on biochemical parameters

Variable	Control	OTA	0.05% A. awamori	0.1% A. awamori	0.15% A. awamori
Total protein (g/dl)	8.19 ± 0.37^a^	7.03 ± 0.33^b^	7.14 ± 0.32 ^a b^	7.43 ± 0.23 ^a b^	7.87 ± 0.21^a^
Albumin (g/dl)	4.05 ± 0.08^a^	3.02 ± 0.06^c^	3.53 ± 0.10^b^	3.67 ± 0.07^b^	3.92 ± 0.09^a^
Glucose (mg/dl)	92.3 ± 5.81^c^	143.32 ± 5.21^a^	124.3 ± 1.50^b^	105.33 ± 5.82^c^	101.72 ± 2.21^c^
AST (IU/L)	54.36 ± 3.52^d^	90.02 ± 2.44^a^	78.44 ± 1.93^b^	65.10 ± 2.03^c^	60.21 ± 2.55^ cd^
LDH (IU/L)	471.72 ± 20.41^d^	1282.09 ± 86.70^a^	900.20 ± 35.15^b^	690.73 ± 40.76^bc^	572.30 ± 22.45^c^
CK-MB	951.74 ± 26.42^c^	2096.30 ± 92.81^a^	1670.03 ± 48.62^b^	1030.15 ± 35.36^c^	991.09 ± 36.17^c^
ALP (IU/L)	17.33 ± 1.75^c^	52.72 ± 3.80^a^	31.37 ± 2.23^b^	22.78 ± 2.03^b^	21.32 ± 1.93^b^
Urea (mg/dl)	39.53 ± 1.74^b^	63.00 ± 3.16^a^	54.75 ± 3.89^a^	42.31 ± 1.54^b^	43.06 ± 2.00^b^
Creatinine (mg/dl)	0.87 ± 0.03^c^	2.07 ± 0.16^a^	1.87 ± 0.15^a^	1.16 ± 0.12^b^	1.12 ± 0.07^b^
Na (mmol/L)	109.03 ± 3.03^a^	87.34 ± 2.81^c^	94.00 ± 2.17^b^	94.12 ± 2.55^b^	94.64 ± 3.82^b^
K (mmol/L)	6.16 ± 0.33^a^	5.09 ± 0.11^c^	5.46 ± 0.25^bc^	5.76 ± 0.42^ab^	5. 96 ± 0.11^a^

Data were presented as mean ± SEM, (*n* = 7 per group)

^a–d^Means within the same row carrying different superscript letters are significantly different at *P* ≤ 0.05. All groups were compared to each other. *Na* sodium, *K* potassium, *TP* total proteins, *Alb* albumin, *AST* aspartate aminotransferase, *LDH* lactate dehydrogenase, *CK*-*MB* creatine phosphokinase, *ALP* alkaline phosphatase

Regarding serum biomarkers concerning cardiac injury, the rabbits fed OTA-contaminated diet exhibited higher enzymatic activities of AST, LDH, CK-MB, and elevated glucose concentration than the control group. Interestingly, the dietary addition of *A*. *awamori* ameliorated nearly all OTA adverse effects with the most significant influence in rabbits who gained *A*. *awamori* at 150 mg/kg diet. Changes in biochemical parameters are illustrated in Table 4.

### Oxidative stress and antioxidant biomarkers

**Fig. 4:**
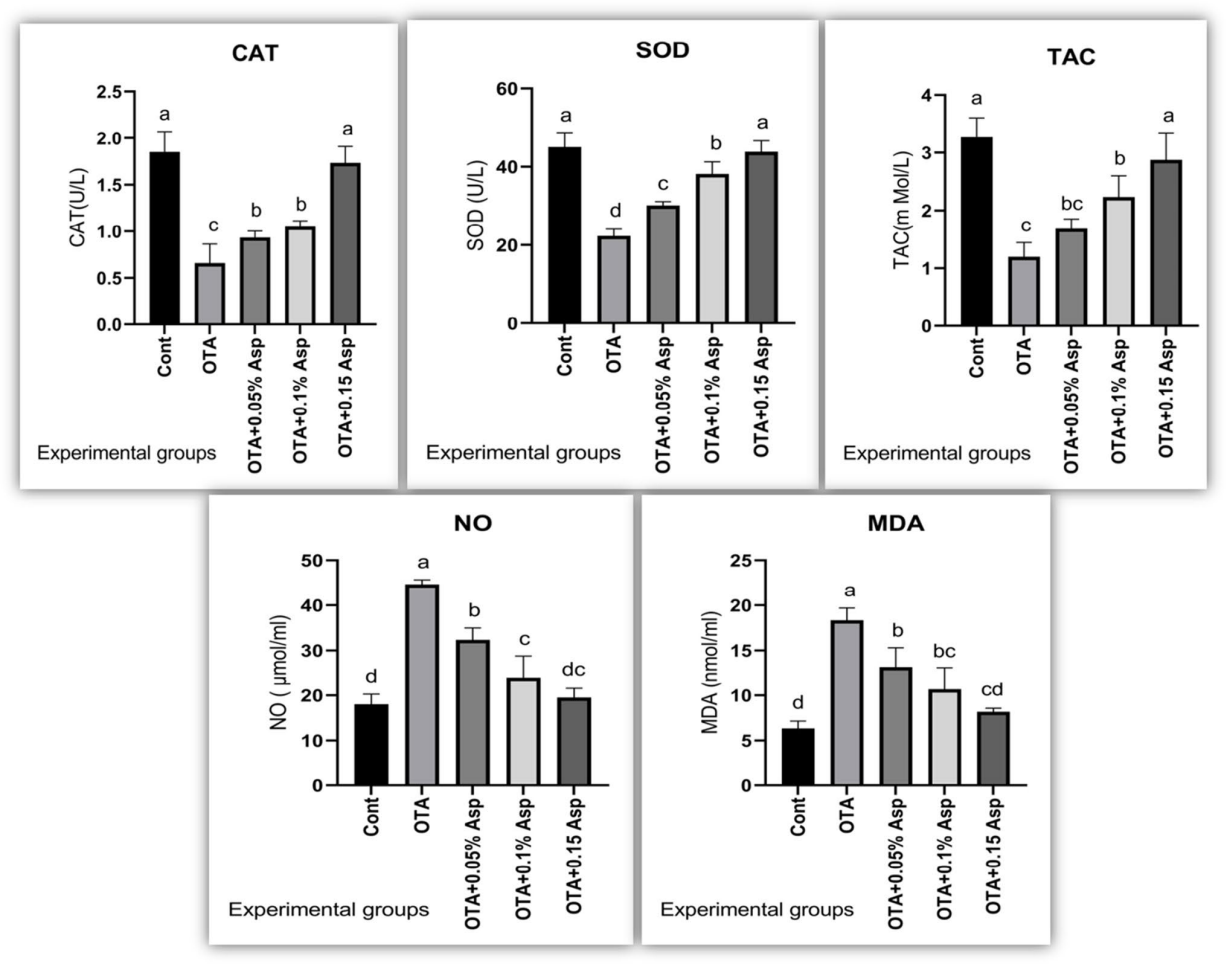
Effect of OTA and/or *A*. *awamori* supplementation on anti-oxidant/oxidant balance. Data were presented as mean ± SEM (*n* = 7 per group). ^a–d^ Means column carrying different superscript letters are significantly different at *P* ≤ 0.05.CAT = Catalase; SOD = Super oxide dismutase; TAC = Total anti-oxidant capacity; NO = Nitric oxide and MDA = Malonalaldehyde

Rabbits intoxicated with OTA exhibited significantly higher serum MDA and NO levels. However, serum TAC, CAT, and Gxp activities were depleted considerably compared with the control rabbit group. *A*. *awamori* dietary supplementation reversed these altered parameters to a comparable level of the control group, with the best effect for *A*. *awamori* at a 150 mg/kg diet. Changes in lipid peroxidation and antioxidant enzymes are portrayed in Fig. 4.

### Histopathological findings of the kidney and heart

#### Kidney

**Fig. 5:**
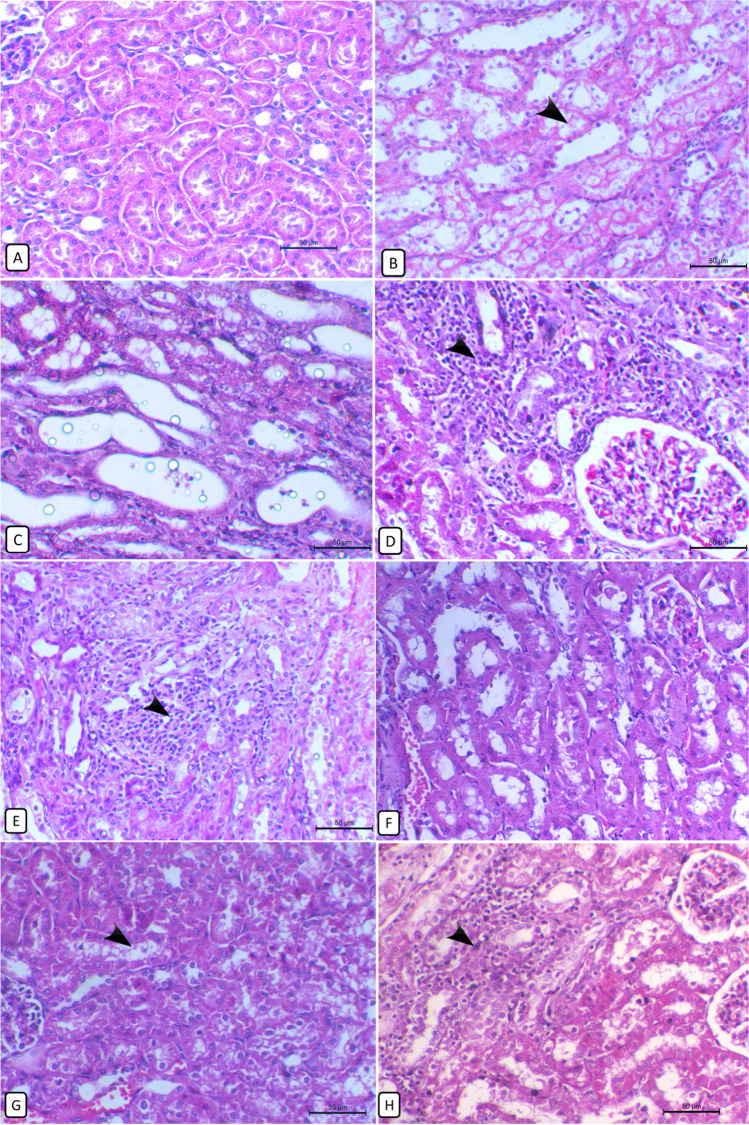
Histopathological changes of the rabbit’s kidney in different experimental groups. **A** Control group shows the normal histological appearance of the kidney. **B** In the OTA-treated group, the kidney shows marked tubular cytoplasmic vacuolation with pyknotic nuclei of renal epithelial cells (arrow).** C** OTA-treated group, kidney showing cystic dilatation of some tubules inducing pressure atrophy of the surrounding parenchyma. **D** OTA-treated group, kidney showing periglomerular mononuclear cells infiltrations *(*arrow*)* predominantly lymphocytes and some heterophils with the presence of a necrotic area of a dark condensed cytoplasm and pyknotic nucleus on the periphery. **E** OTA-treated group, kidney showing focal interstitial mononuclear cells infiltrations (arrow) mainly of macrophages and lymphocytes accompanied with variable degenerative changes of the surrounding tubules. **F** OTA + 150 mg/kg diet of *A*. *awamori*-treated group, kidney showing marked restoring of normal renal architecture with mild inter-tubular vessels dilation and few renal casts. **G** OTA + 100 mg/kg diet of *A. awamori*-treated group, kidney showing a moderate degree of renal damage with vacuolar tubular degeneration *(*arrow*)* and some necrotic tubules. **H** OTA + 50 mg/kg diet of *A*. *awamori*-treated group, kidney showing marked interstitial inflammatory cells infiltration (arrow) and an area of tubular necrosis. All are H&E stained, bar = 50 µ

The kidneys of control rabbits showed the normal histological appearance of the glomeruli and renal tubules in both cortical and medullary regions (Fig. 5A). In contrast, rabbits provided with OTA exhibited various histological alterations; hyperemia in glomerular capillaries and interstitial vessels and marked vacuolar degeneration of tubular epithelium (Fig. 5B). Other renal tubules showed cystic dilatation with pressure atrophy on the surrounding tubules (Fig. 5C). In addition, multiple focal mononuclear cell infiltrations, mainly of macrophages and lymphocytes with some heterophils, are either periglomerular (Fig. 5D) or interstitial (Fig. 5E). In addition, focal areas of coagulative tubular necrosis were noticed either cortically or medullary with a condensed eosinophilic cytoplasm and pyknotic nucleus. Also, hyaline casts in some of the tubular lumens were seen. The severity of these tubular lesions was improved by supplementation of *A*. *awamori* in a dose-dependent response with the highest restoration of renal parenchyma at a concentration of 150 mg/kg of *A*. *awamori* (Fig. 5F) with minimal degenerative changes. A moderate alleviation of renal histological changes was observed at a concentration of 0.1℅ with a modest degree of renal tubular vacuolation, necrosis, and cellular infiltrations (Fig. 5G). However, in rabbits supplied with a 50 mg/kg diet of *A*. *awamori*, a mild defending effect against the OTA renal damaging effect (Fig. 5H) with continuity of tubular degenerative changes and inflammatory cells infiltration.

#### Heart

**Fig. 6:**
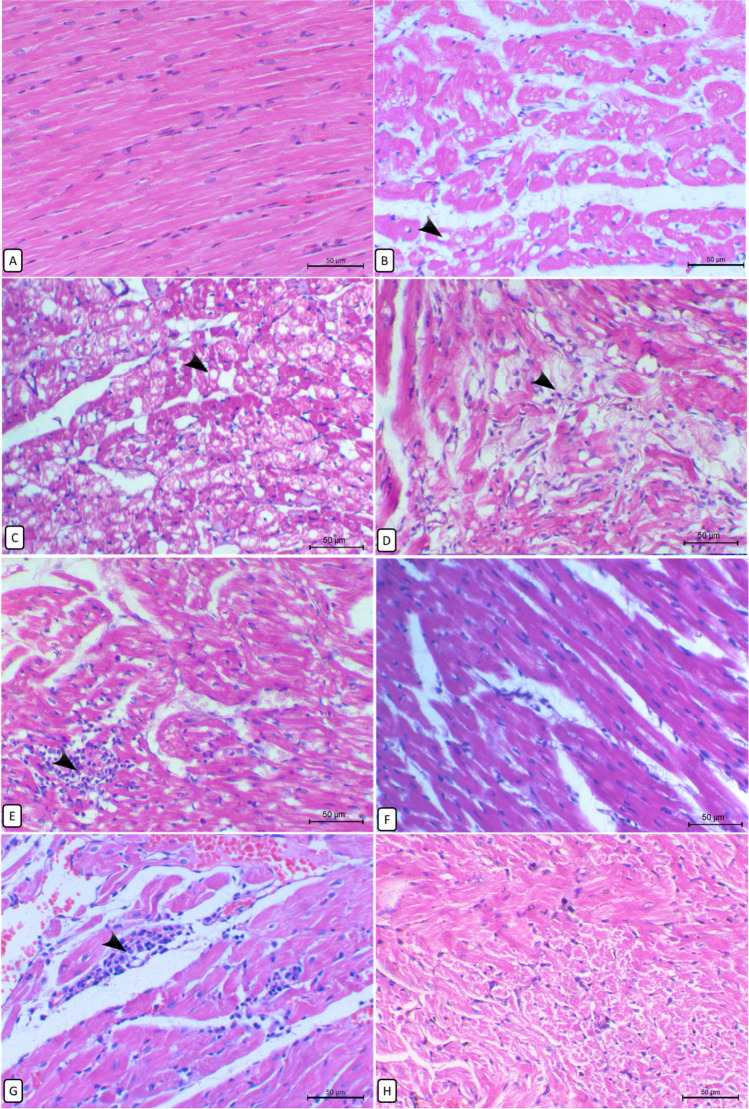
Histopathological changes of the rabbit’s heart in different experimental groups. **A** Control group, normal histological structure of the heart. **B** OTA-treated group, heart showing fatty vacuolation (arrow) of cardiac myocytes with variably sized vacuoles (arrow). **C** OTA-treated group, heart showing massive degenerative changes of myofibers with cytoplasmic rarefaction and loss of nuclei (arrow) and others showing cell necrosis with eosinophilic cytoplasm and pyknotic nuclei. **D** OTA-treated group, heart showing muscle degeneration and loss of myocardial bundles arrangement (arrow) with slight infiltration of mononuclear cells and atrophy of the surrounding myocytes. **E** OTA-treated group, heart showing the focal area of mononuclear cells infiltration (arrow) in between muscle fibers with disorganization of the surrounding myofibers. **F** OTA + 0.15% *A*. *awamori*-treated group, heart showing marked improvement of the heart histological appearance where no cytoplasmic vacuolation or swelling. **G** OTA + 0.1% *A*. *awamori*-treated group, heart showing moderate degenerative changes of myocardial cells accompanied with slight inflammatory cells infiltration (arrow) and areas of hemorrhage. **H** OTA + 0.05% *A*. *awamori*-treated group, heart showing marked myolysis with rarefied cytoplasm and karyolysis. All are H&E stained, bar = 50 µ

The heart of the control group showed a normal histological appearance of regularly distributed myocardial fibers (Fig. 6A). In contrast, hearts of the OTA-exposed group showed marked swelling of myocardial fibers, marked fatty vacuolation of myocardial cells (Fig. 6B) in some parts of the myocardium, and others showed complete myolysis with rarefied cytoplasm and karyolysis (Fig. 6C). The degenerative changes were exaggerated to necrosis and destruction of the muscle fibers (Fig. 6D) and loss of their regular distribution pattern. Focal infiltration of mononuclear cells between the muscle fibers (Fig. 6E) was detected in addition to multiple areas of vascular congestion and hemorrhages. Amazingly, in rabbits exposed to combine administration of OTA and *A*. *awamori*, there was a notable improvement in heart structure which was more distinct in rabbits supplemented with a 150 mg/kg diet of *A*. *awamori*, where no cytoplasmic vacuolation or swelling of myocardial cells was seen (Fig. 6F). In rabbits supplied with a 100 mg/kg diet of *A*. *awamori*, the degree of congestion, myocardial cytoplasmic vacuolation, necrosis, and inflammatory cell infiltration was moderately decreased (Fig. 6G). Unfortunately, the hearts of rabbits that received a 50 mg/kg diet of *A*. *awamori* were weakly restored the normal histology showed degenerative and necrotic changes (Fig. 6H).

### Gene expression of oxidative stress and inflammation-related genes

**Fig. 7:**
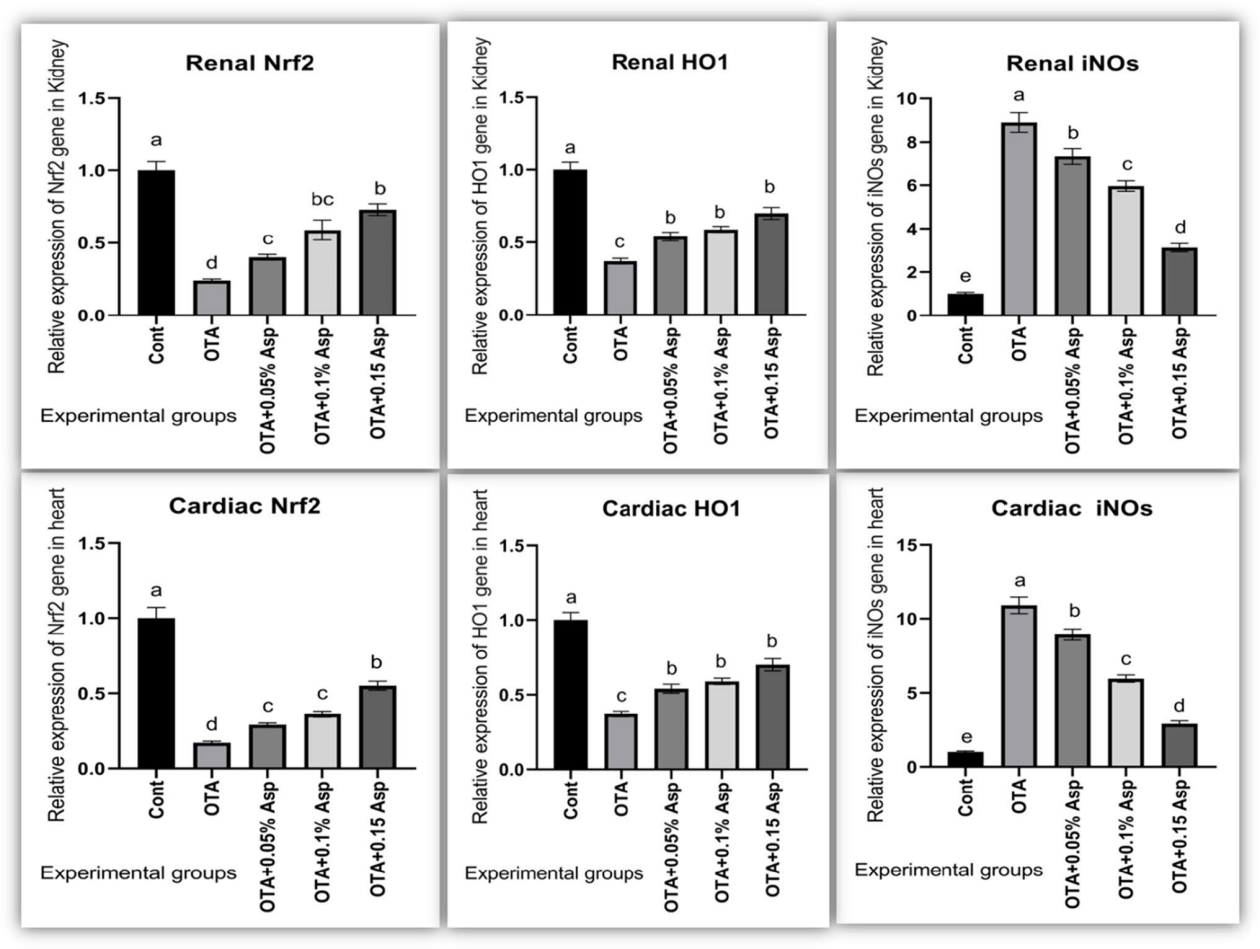
Influence of OTA and/or *A*. *awamori* administration on Nrf2, HO-1, and iNOS mRNA expression in renal and cardiac tissues of rabbits. Data were presented as mean ± SEM (*n* = 7 per group). ^a−d^ Means coulmncarrying different superscript letters are significantly different at *P* ≤ 0.05. *Nrf2* nuclear factor E2-related factor 2, *HO*-*1* heme oxygenase-1, *iNOS* inducible nitric oxide synthase

**Fig. 8:**
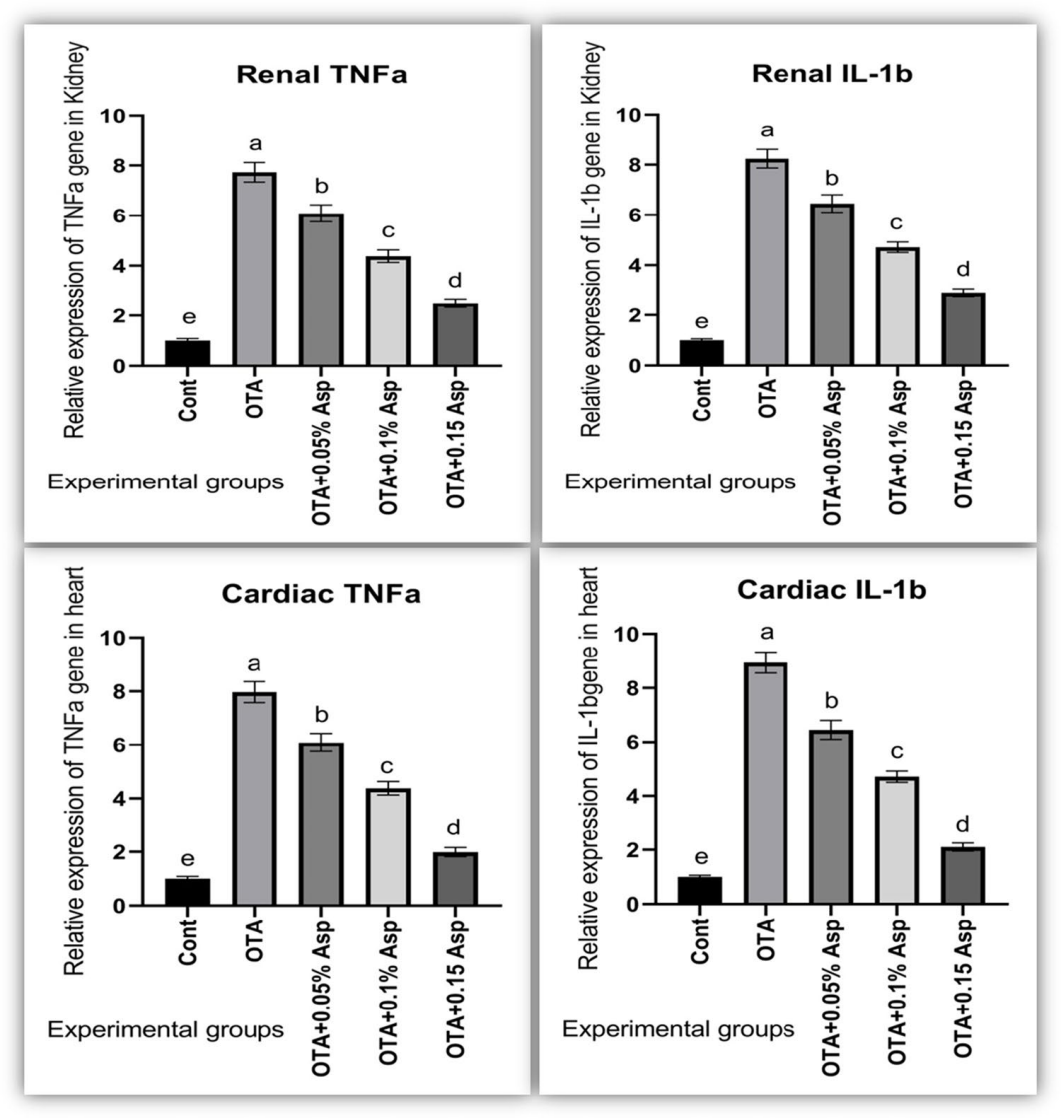
Influence of OTA and/or *A*. *awamori* administration on IL-1β and TNF-α mRNA expression in renal and cardiac tissues of rabbits. Data were presented as mean ± SEM (*n* = 7 per group). Transforming growth factor alpha = TNF-α and Interleukin 1 beta = IL-1β. ^a–d^ Means coulmncarrying different superscript letters are significantly different at *P* ≤ 0.05

In OTA fed rabbits, significant downregulation of mRNA expression level of Nrf2 and HO-1 (cytoprotective factor regulating the expression of genes coding for antioxidant) genes was seen while marked upregulation of iNOs (nitrosative stress) level (Fig. 7) along with TNF-α and IL-1β expression levels (Fig. 8). The dietary addition of *A*. *awamori* modulated these gene expression profiles. The best regulatory effect was correlated with *A*. *awamori* at a 150 mg/kg diet.

## Discussion

Many reports demonstrated the adverse toxicological influences of OTA on rabbits (Mézes 2008; Sun et al. 2018; Jedziniak et al. 2019). Here, the rabbits fed a diet contaminated with OTA showed a mortality rate of about 16.67%, similar to data reported by other researchers (Kumar et al. 2003; Elaroussi et al.^2006^). Moreover, OTA reduced feed intake, body weight gain, and elevated FCR, indicating poor growth performance (Zhang et al.^2016^ and Gan et al. 2017). The reduction in weight gain may be due to the harmful effect of OTA on the intestinal tract by reducing feed absorption (Raju and Devegowda, 2000). Unlike rabbits fed a diet contaminated with OTA alone, those cotreated with *A*. *Awamori* showed improvement in growth performance following (El-Deep et al.^2020^). This improvement may be attributed to the endogenous release of digestive enzymes in the animal’s gut, which accelerates the digestion and absorption of ingested diets (Phuoc and Jamikorn, 2017). In addition to increasing the metabolizable energy of the feed along with enhancement of cellulose digestion, volatile fatty acid formation or due to enhancing the survival and implantation of live microbial content in the gastrointestinal tract (Saleh et al. 2012) or duo the improvement of lipid and fiber digestibility (El-Deep et al. 2020). Broilers fed on diets containing 0.05% and 1% *A*. *Awamori* increased the carcass weights (Yamamoto et al. 2007). Cellulase and xylanase enzymes are required for the digestion of soluble non-starch polysaccharides that were found to be produced by *A*. *awamori* (Bhat and Hazlewood 2001). Aspergillus is known to improve the nutritional quality of soybean due to its enzymes that degrade trypsin inhibitors (Hong et al. 2004). A growth-promoting effect was found in fermented products processed by *A*. *awamori* (Kamizono et al. 2013); *A*. *awamori* was also involved in the appetite regulation in the central nervous system (Kamisoyama et al. 2009).

OTA harms the immune response and antioxidant status, which ultimately increases the susceptibility of animals to metabolic diseases and tissue damage (Kumar et al. 2014). OTA has an immunosuppressive effect as a potent inhibitor of protein synthesis, which delays the division of the rapidly dividing cells of the immune system (Harvey et al. 1992). OTA impairs lymphocytes production, activation, differentiation, and proliferation (Haubeck et al. 1981; Lea et al. 1989). Several in vitro studies focused on OTA impacts on neutrophils and macrophages, including oxidative stress, apoptosis, phosphorylation of the ERK1/2, and release of TNF-α via NF-kB pathways (Liu et al., 2012; Giromini et al. 2016; Brennan et al. 2017). OTA also decreases the phagocytic activity of the macrophages, probably due to the reduction of basal interferon (Harvey et al. 1992). In the present study, dietary *A*. *awamori* improved the suppressed immune response induced by OTA. A more robust immune response was indicated by the increased total leukocyte and lymphocyte counts and increased phagocytic activity and phagocytic index of heterophils with elevated complete proteins concentrations. Our results agreed with Falcao-e-Cunha et al. (2007), who reported that prebiotics stimulates the immune responses in rabbits.

Additionally, Sakai et al. (2001) recorded enhancement of phagocytic activity following the addition of yeast to OTA-intoxicated Nile tilapia. Similarly, Mateos et al. (2010) detected a stimulatory effect on rabbits’ immune response when fed a diet with specific oligosaccharides. Yang et al. (2009) stated that probiotics could enhance the immune system by adhering to the intestinal mucosa. Further, probiotic stimulates the immune response by activating macrophages and lymphocytes and higher production of immunoglobulins and γ-interferon. Similarly, Wintrobe (1983) who recorded increase in the total leukocyte count by probiotic supplementation duo to polymorphonuclear cell proliferation in the white pulp with activated B-lymphocytes in the red pulp of the spleen.

Moreover, probiotic plays a pivotal role in inducing a healthy intestinal structure and enhancing immunity Rajput and Li (2012). Additionally, Glick (2000) stated that gut microflora is essential for the early stimulation and maturation of the cellular component of the intestinal immune system. Therefore, an improved intestinal microenvironment stimulates pluripotent hemopoietic precursors into clones of lymphocytes, which could be one factor in the increase in total leukocyte count.

Oxidative stress is an important mechanism associated with the OTA-induced nephrotoxicity. Therefore, we measured the oxidative and nitrosative stress markers MDA and NO together with antioxidant biomarkers TAC, CAT, and SOD activities. *A*. *awamori* at the studied concentrations exerted antioxidant power in a dose-dependent response. In our study, the protective effect of *A*. *awamori* against OTA-induced oxidative stress could be either directly by scavenging free radicals and inhibiting lipid peroxidation by reducing MDA and NO levels or indirectly via enhancing the antioxidant activity of SOD, CAT, and TAC. *A*. *awamori* owed higher antioxidant active constituents such as (citric acid, citric acid isomers, octadecenoic, octadecadienoic, and octadecanoic acid's derivatives), polyphenols, and flavonoids that potentiate *A*. *awamori* biological activity as antioxidant and anticancer agents (Assar et al. 2021). Lin et al. (2012) stated that *A*. *awamori* increased antioxidant activity and DNA protecting effect concerning the conversion of phenolics of litchi pericarp, a readily accessible source of the natural antioxidants in the food industry. Similarly, Lee et al. (2008) stated that *A*. *awamori* was superior for exhibiting the highest antioxidant activity during the fermentation process due to polyphenol content enhancement of the β-glucosidase enzyme. El-Deep et al. (2014; 2021) and Abdelhady et al. (2017) provided an additional explanation for *A*. *awamori* antioxidant activity through increasing mRNA expression of genes encoding antioxidant enzymes such as GPx, SOD, and CAT in broiler chickens and rabbits fed a diet with *A. awamori*. Saleh et al. (2012) and El-Deep et al. (2014) reported that the dietary addition of *A*. *awamori* (0.1%) or *A*. *niger* (0.05%) or feeding *A*. *awamori* (0.05%) declined the thiobarbituric acid reactive substances (TBARS) value while enhanced α-tocopherol content in the broiler breast muscle referring to *A*. *awamori* antioxidative properties.

The current work showed that rabbits who received OTA suffered from normocytic normochromic anemia and leukocytosis, neutrophilia, monocytosis, lymphopenia, eosinopenia, and increased H/L, indicating a stress picture of leukogram. These results supported the findings of Elaroussi et al. (2006) and Sawale et al. (2009), who detected a significant reduction in the total number of erythrocytes in broiler chickens fed an OTA-contaminated diet. The high number of WBCs despite the declined RBCs, Hb, and PCV indicates a partial depressive effect of OTA on the bone marrow duo metabolic disturbance (Janaczyk et al. 2006). H/L is disturbed by stressors, and it can be used as a sensitive hematological sign of stress response (Janaczyk et al. 2006; Kowalski et al. 2006 and Salamano et al. 2010). Jelkmann (2011) stated that multiple mechanisms might contribute to anemia in renal diseases; tubular atrophy during chronic kidney disease generates tubule-interstitial fibrosis, leading to a reduction of erythropoietin synthetic capacity contributes to the associated anemia. In addition, the increase of serum alkaline phosphatase and hyperphosphataemia may also play a role in chronic renal disease-associated anemia and EPO hypo-responsiveness and reduction in erythropoietin hormone (Epo) expression (Stachurska et al., 2013). Simultaneous dietary supplementation with *A*. *awamori* can reverse the OTA-induced reduction in total RBCs, PCV%, and Hb concentration. In harmony with Fathi et al. (2017), who detected that *A*. *awamori* as a probiotic enhanced the RBCs count and hemoglobin concentration in rabbits via its anti-inflammatory and immune-modulatory properties (Group 2014). This may pinpoint the free radical-scavenging properties and the antioxidant activities of *A*. *awamori* (Assar et al., 2021) that protect against lipid peroxidation of the erythrocyte membrane.

OTA induces oxidative stress and releases reactive oxygen and nitrogen species (Tao et al., 2018) therefore mediating cellular damage with the release of intracellular enzymes (Surai et al., 2008). In the current study, we found a remarkable increase in levels of creatinine, urea, and ALP activities with the marked decline of sodium, potassium, total proteins, and albumin levels in OTA-intoxicated rabbits as compared to the control rabbits. Although exact mechanisms involved in OTA-induced renal toxicity are not fully known, Glahn et al. (1989) suggested that OTA may cause an osmotic diuresis in pullets by inhibiting the tubular reabsorption of electrolytes. Anzai et al. (2010) stated that OTA mainly impairs proximal tubular functions and causes glucosuria. The elevated urea and creatinine levels could be attributed to the inhibiting effect of OTA metabolites on glomerular filtration and catabolic protein rates, which reduced the renal capacity to get rid of urea (Akturk et al., 2006). Serum total proteins concentrations are commonly used as humoral immunity indicators. The reduced level of complete serum proteins can be due to vascular leaking and failure of their synthesis, and elevated rates of proteolysis. The reduced albumin concentration can be explained by increased renal excretion and loss of protein synthesis by OTA-induced liver malfunction in rabbits (Kumar et al., 2007). The noted hypoproteinemia may also be attributed to insufficient digestion; malabsorption preceded by damage to the gastrointestinal tract. Mycotoxins can also exert their effects on the hepatic tissue causing hepatocytes lipid infiltration, necrosis, or hepatic cell death (Cagauan et al. 2004). ALP can be drastically increased in patients with renal insufficiency and duo hepatic injuries. OTA mediated kidney damage through induction of oxidative stress that arises from the excessive generation of ROS, which has been reported to attack various biological molecules, including lipids, and causes lipid peroxidation (Zheng et al. 2013). Kamp et al. (2005) reported that OTA induces oxidative stress, which may lead to subsequent damage or initiation of the apoptotic process. Increased apoptosis rates in the kidney may lead to polycystic kidney disease, glomerular sclerosis, or interstitial fibrosis. Klaric et al. (2008) showed that OTA induced cytotoxicity and apoptosis in porcine kidney 15 (PK15) cells, suggesting that OTA-induced nephrotoxicity is related to oxidative damage. However, renal dysfunction biomarkers were significantly modulated by *A*. *awamori* supplementation, suggesting its improving effects on rabbits (El-Katcha et al. 2011).

Cardiac injury biomarkers such as CK-MB and LDH are markedly released into the extracellular fluid during cardiac damage. In this experiment, OTA-treated rabbits exhibited markedly elevated enzymatic activities of AST, LDH, and CK-MB together with increased glucose concentration as compared with the control group. OTA-treated group revealed elevation of all myocardial enzymes (Cui et al. 2020) due to mitochondrial dysfunction. Similarly, reported biochemical changes in chickens’ heart injury induced by OTA. Moreover, OTA hyperglycemic effect in the exposed rabbits may be due to a disturbance in carbohydrate metabolism Verma and Shalini (1998). Verma and Shalini (1998) supported our results, reporting that OTA could induce hyperglycemia in rabbits. Simultaneous dietary *A*. *awamori* counteracts the OTA hyperglycaemic effect. Consistent with this, Takemoto et al. (2014) found that *A*. *awamori*-fermented diet ameliorated the alloxan-induced hyperglycemia in mice.Similarly, Doi et al. (2015) confirmed that Burdock Root fermentation with *A*. *awamori* inhibited the induced hyperglycemia.

Here, the observed renal histopathological alterations due to OTA exposure were consistent with previous reports by Battacone et al. (2010). OTA can induce degenerative and necrotic lesions in the kidneys (Francisco and Maria, 2010), and these lesions could be correlated to oxidative damage of OTA. Likewise, retardation of growth performance with renal histopathological changes was also observed in piglets after OTA exposure (Zhang et al. 2016 and Gan et al. 2017), rabbits (Prabu et al. 2013), rats (Aydin et al. 2003; Biro et al. 2002 and Malekinejad et al. 2011), and porcine through inducing renal cytotoxicity and apoptosis (Klaric et al. 2008). The observed cystic dilation in OTA-treated rabbits may be attributed to the direct toxic effect of OTA on the tubule epithelium impairing their absorption and secretion, or may result from the lower urinary tract obstruction, deposition of tubular crystals, interstitial inflammation and/or fibrosis, and chronic progressive nephropathy (Greaves 2012). Moreover, cystic dilatation was also observed in nephropathic mice exposed to dietary OTA at 40 μg/g (Wanda et al. 2002). The observed cast formation may be owed to the tubular epithelium detachment, deposition, and interaction with the tubular lumen proteins. Secondly, impaired sodium reabsorption because of damaged tubular epithelium, which increases sodium concentration in the tubular lumen, producing protein polymerization (Abuelo 2007). Rising renal injury biomarkers supported these findings as urea, creatinine, and ALP and elevation of oxidative stress markers in line (Jan et al., 2017). In this work, OTA-intoxicated rabbits exhibited marked myocardial histopathological changes confirmed by the remarkable increase in serum cardiac injury biomarkers and the notable elevation of serum oxidative stress biomarkers such as MDA and NO with significant inhibition of TAC, CAT, and GPX activities. In harmony with, who reported that OTA induced chickens’ heart injury biomarkers elevation. noted that OTA induced myocardial congestion, swelling and necrosis, and ultrastructural changes in the myocardium of rats. Similar histopathological changes were reported in OTA-treated rats (Hussein et al. 1997). In the same line, Cui et al.(2020) reported that OTA-induced mouse myocardial tissue damage was represented by massive cytoplasmic vacuolar degeneration, myocardial swelling, and necrosis due to mitochondrial dysfunction. Stoev et al. (2021) detected some lytic changes and irregular staining of the myofibrils of OTA exposed chicks. OTA strongly affects the performance of the myocardium and the cardiovascular system of the rat. The OTA-induced myocardial injury may be due to the elevated calcium level within the myocardium (Hussein et al., 1997). Khan et al. (1989) suggested a possible direct effect of OTA on the integrity of myocardial cell membrane through leakage of calcium-loaded microsomes resulting in an influx of extracellular calcium ions. Interestingly, dietary *A*. *awamori*-supplied rabbits restored the altered renal and cardiac histological architecture to normal in a dose-dependent response with the best improvement at the highest concentration of *A*. *awamori*.

NO production is increased in inflammation and has pro-inflammatory and regulatory effects. Cavin et al. (2009) reported that OTA stimulated NO’s formation through an NF-kB-dependent induction of iNOS. Here, we also found higher expression levels of *IL1b*, *TNFa*, and *iNOS* in the kidney and hearts of OTA intoxicated rabbits. The mechanism of NO stress and genotoxicity may be explained by its reaction with superoxide radical to form the pro-oxidant peroxynitrite, which rapidly decomposed to the nitro radical, causing nitrosative stress and DNA damage (Heikal et al., 2009). All these findings suggest the involvement of oxidative stress in the OTA-mediated renal and cardiac toxicity and point out the role of ROS in OTA’s adverse effects. In the present experiment, OTA-intoxicated rabbits exhibited a significant reduction in renal and cardiac mRNA expression of Nrf2 and HO-1 with a marked elevation in renal and cardiac mRNA expression of iNOS compared with the control group. Boesch-Saadatmandi et al. ( 2008) reported that OTA nephrotoxic effect was mediated by the downregulation of Nrf2 and *HO-1* genes. Similar findings for reducing Nrf2 and HO-1 were detected in the kidneys of piglets (Marin et al., 2018) and the myocardial tissue of mice treated with OTA (Cui et al., 2020). HO-1 is recognized for its cytoprotective activities, chiefly in the cardiovascular system, even though its positive impacts on kidney diseases have been described in several diverse in vitro and animal models (Jarmi and Agarwal 2009). Surprisingly, stated that complicated mechanisms of OTA nephrotoxicity might be partially overcome by HO-1 activation through upregulation of MAPK kinases activity via inducing ERK1/2 and activation of major Nrf2-regulated antioxidants enzymes. Nrf2 participates in both the basal expression and induction of several genes, including genes encoding for detoxification, cytoprotection, and antioxidant enzyme activation (Kensler et al., 2007). OTA also inhibited SOD increased ROS, and weakened the expression of glutathione S-transferase (GST) with reduced activation of Nrf2 (Bösch-Saadatmandi et al., 2009). Herein, inhibition of Nrf2 is strongly linked with the inhibition of HO-1; lowered levels of serum TAC, CAT, and GPX activities; and the observed renal and cardiac histopathological alterations of OTA-exposed rabbits. Therefore, inhibition of antioxidative defense is the probable mechanism that could contribute to OTA nephrotoxicity and cardiotoxicity. Interestingly, OTA-intoxicated rabbits supplied with dietary *A*. *awamori* upregulated Nrf2 and HO-1 in a dose-dependent response. Thus, *A*. *awamori* reversed the oxidative stress by achieving the balance between liberating (MDA and NO) and scavenging ROS by (TAC, CAT, and SOD) through inducing (Nrf2 and HO-1) expressions. It is well-known that Nrf2 activators potentially hinder carcinogenesis in animal models and humans (Cavin et al., 2007). Nrf2 plays a role in protecting cells from oxidative stress and inflammatory insults (Chen et al. 2006). Similarly, found that selenium-rich yeast attenuated OTA-induced small intestinal injury in broilers by activating the Nrf2 pathway and inhibiting NF-KB. The inflammatory response is organized by pro-inflammatory cytokines such as IL-1β, IL-6, and TNF-α. Furthermore, IL-lβ triggered the expression of iNOS mRNA, which stimulated NO production. Our results have shown that OTA induces a significant increase of IL-1β and TNF-α mRNA in the kidney and heart of intoxicated rabbits, referring to an OTA-induced inflammatory response. Marin et al. (2017 and 2018) observed higher levels of IL-1β and TNF-α in piglets’ kidneys sub-chronically intoxicated with OTA. In contrast, the dietary addition of *A*. *awamori* to OTA-intoxicated rabbits has an anti-inflammatory effect via downregulation of IL-1β and TNF-α. The hepatoprotective effect of *A*. *awamori* against aflatoxin B1 induced hepatic oxidative stress and inflammation in rabbits was previously investigated by Abdelhady et al. (2017) and against the initiation process of liver carcinogenesis caused by diethylnitrosamine (DEN) in a rat model (Assar et al., 2021).

## Conclusions

This study revealed that the exposure of growing rabbits to an OTA-contaminated diet retarded their growth, altered immune-hematological and biochemical parameters, and induced histopathological changes in the kidney and heart of the exposed rabbits. To the best of our knowledge, this is the first report which pinpoints the protective effect of simultaneous dietary supplementation of *A*. *awamori* against OTA-induced renal and cardiac injuries through potentiating the rabbit’s anti-oxidant defense system via inducing the inducing Nrf2 signaling pathway, which activates heme oxygenate-1 (HO-1) enzyme, therefore enhancing TAC, CAT, and SOD activities and reducing oxidative and nitrosative stress by downregulating of iNOS expression thus reducing MDA and NO levels. In addition to its anti-inflammatory properties via downregulating of IL-1β and TNF-α expression levels, the best improvement at the highest concentration of the supplied *A*. *awamori*. Based on our observations, *A*. *awamori* could be utilized as a natural nephron and cardio protective agent against ochratoxicosis in rabbits.

## Data Availability

The authors confirm that the data supporting the findings of this study are available within the article.

## Abbreviations

*A*. *awamori*: Aspergillus awamori
AST: Aspartate aminotransferase
ALP: Alkaline phosphatase
CAT: Catalase
CK-MB: Creatine kinase-MB
TNF-α: Tumor necrosis factor-alpha
IL-1β: Interleukin 1 beta
HO-1: Haemoxygenase-1
iNOS: Nitric oxide synthases
LDH: Lactate dehydrogenase
Nrf2: Nuclear factor erythroid 2-related factor 2
MDA: Malondialdehyde
NO: Nitric oxide
OTA: Ochratoxin A
SOD: Superoxide dismutase
TAC: Total antioxidant capacity
